# Use of the Generalized Vector Addition Theorem for Antenna Position Translation for Spherical Mode-Filtering-Based Reflection Suppression

**DOI:** 10.3390/s25175557

**Published:** 2025-09-05

**Authors:** Marc Dirix, Stuart F. Gregson, Rostyslav F. Dubrovka

**Affiliations:** 1 E&C Anechoic Chambers, 2260 Westerlo, Belgium; md@ecac.be; 2 Institute of High Frequency Technology, RWTH-Aachen, 52074 Aachen, Germany; 3 Next Phase Measurements, 11521 Monarch St, Garden Grove, CA 92841, USA; 4 School of Electronic Engineering & Computer Science, Queen Mary University of London, Peter Landin Building, London E1 4NS, UK; r.dubrovka@qmul.ac.uk

**Keywords:** antenna measurement, echo suppression, spherical wave expansion, vector addition theorem, mode filtering

## Abstract

**Highlights:**

**What are the main findings?**

- Unlike other perhaps better-known techniques, the generalized vector addition theorem can be successfully utilized to perform antenna position translations in any direction, and for displacements that can be smaller or larger than the maximum radial extent of the antenna;
- Crucially, this paper shows how the all-important vector addition coefficients can be obtained from the scalar coefficients for which efficient, accurate, and precise recurrence relations exist, which greatly reduces the computational effort required, which would otherwise likely render the technique impractical.

**What is the implication of the main finding?**

- The technique presented provides the first-ever algorithm for applying mode-filtering-based reflection suppression without the need to first transform to the asymptotic far-field, yielding a significant generalization of the algorithm;
- The new algorithm represents a notable development as it is rigorous and general, incorporating both reactive and propagating components, thereby making the processing applicable to a wider range of problems than has previously been the case.

**Abstract:**

Monochromatic mode-filtering-based scattering suppression techniques have been shown to be applicable to all commonly used forms of far- and near-field antenna and RCS measurement techniques. Traditionally, the frequency-domain mode-filtering technique takes a far-field pattern, either measured directly or obtained using a suitable near-field to far-field transformation, as its starting point. The measurement is required to be conducted such that the antenna under test (AUT) is positioned offset from the origin of the measurement coordinate system. This physical offset introduces a phase taper across the AUT pattern and results in far greater interference occurring between the direct and indirect parasitically coupled spurious scattered signals. The method is very general and can be applied to all forms of near- or far-field measurements. However, for the case of a spherical near-field measurement (SNF) approach, it is somewhat cumbersome and tedious as first we must perform a probe-corrected spherical near-field to far-field transformation, which itself involves the computation of a complete set of spherical mode coefficients, and then after the displacement has been applied to the far-electric-fields, a second spherical wave expansion and summation is required to implement the mode-filtering procedure. While this data processing chain has been widely deployed and exhaustively validated, it requires passing through the asymptotic far-field, which inevitably results in additional computational effort, as well as incurring some loss of information, which can impose limitations on further near-field applications. This paper introduces an alternative, novel, rigorous algorithm that applies the displacement of the AUT directly using the vector addition theorem for spherical waves. An efficient implementation has been developed, and it is shown that the new, rigorous algorithm for the translation and filtering can be easily implemented directly within the data processing chain of any standard spherical near-field transformation algorithm, avoiding the need to first transform to the asymptotic far-field and also removing the need for a secondary spherical mode expansion and secondary spherical mode summation. While the vector addition theorem required for the spherical near-field to far-field transformation (SNFFFT) algorithm has been described in detail in the open literature, its implementation has been limited to the case of impinging waves and positive *z*-directed translations where the magnitude of the displacement is necessarily larger than the minimum sphere radius (MRE). In the current paper, the addition theorem will be derived in a new form that allows the translation to be applied in *any* desired direction, without the need for additional rotations, as well as being valid for solutions for waves transitioning through the sphere and applicable for the case where the magnitude of the translation is smaller *or* larger than the radius of the minimum sphere.

## 1. Introduction

Typically, when acquiring indoor antenna measurements, spurious parasitically coupled scattered fields are largely attenuated by covering the interior of the chamber and much of the measurement apparatus with RF-absorbing material (RAM). This material is most often formed from open-cell, carbon-impregnated foam. While these types of absorbers are often considered to be broadband in nature, band limitations do exist, with these being largely governed by their electrical and physical size. The physical size is strongly coupled to their economic cost at the lower end of the spectrum and manufacturing precision at the higher end. This material cannot be perfectly matched to illuminating fields incident from all possible directions and polarizations, with the inevitable impedance mismatch resulting in some form of scattering. Furthermore, whilst considerable time, trouble, and ingenuity can be devoted to optimizing the placement of this absorbent material within the test range, it is not possible to install this on every surface, and generally, linear bearings, lights, fire detection and suppression equipment, etc., are often left exposed. This has become more of an issue in recent times with the rapidly increasing deployment of industrial multi-axis robots for test antenna and probe positioning. Here, the task is further complicated since absorber placement can serve to limit the final available range of motion, which is undesirable in a system that may routinely switch between several very different antenna acquisition modes. Thus, recourse to post-processing-based techniques to improve the quality of the antenna and scattering measurements becomes desirable in a wide range of test and measurement applications.

Although many scattering suppression methods have been proposed, with examples including time-gating (both hardware and software), spatial filtering, RF background subtraction, parametric repeat measurements, and waveform correlation, it is only during the past two decades that the use of mode orthogonalization and filtering-based post-processing techniques have become widely deployed in industry and academia to identify and subsequently extract measurement artifacts arising from parasitically coupled spurious scattered fields in antenna pattern and radar cross-section (RCS) measurements [1,2,3,4,5,6]. These have been used very successfully to greatly improve the facility-level uncertainty budget [7] and have gradually become available for use with all commonly encountered forms of near- and far-field antenna range measurements.

These single-frequency mode-filtering-based approaches have been extended to admit the possibility of processing data that are sampled on irregular grids [6] and, more recently, have been extended to take advantage of sparse sampling compressive sensing techniques [8,9]. Underpinning the success of each of these reflection suppression post-processing strategies and other modes of orthogonalization and filtering strategies is the behavior of the orthogonal vector wave (mode) expansions that are utilized to describe the radiated free fields and, in particular, their behavior under the isometric coordinate translations that are central to the success of the post-processing [6]. Hitherto, all such translations have been implemented by first obtaining the true or quasi-far-fields. A differential phase change has then been applied to those fields to mathematically translate the test antenna back to the origin of the measurement coordinate system before those fields are re-expanded onto a convenient set of basis functions, i.e., modes, so that the mode filtering can be applied [6]. Traditionally, the modal basis has been, respectively, either cylindrical or spherical, depending largely upon whether one- or two-dimensional data are being considered. However, the need to transform to the far-field to implement the translation of origins necessarily attenuates any evanescent fields present and, for the spherical near-field case, requires an additional spherical mode expansion and a further additional spherical mode summation that in principle is unnecessary if instead the translation of origins operation were performed *directly* upon the spherical mode coefficients (SMC) by instead harnessing a generalized vector addition theorem for the isometric antenna position translation.

Standard spherical near-field theory is based on the transmission equation derived by Jensen and Wacker [10,11], where the AUT and the probe are described by spherical mode coefficients that are the complex coefficients of basis functions that are themselves elementary solutions to Maxwell’s equations in a spherical coordinate system. In principle, the transmission equation is valid for any arbitrary test antenna and probe combination at any orientation and separated by a distance between the spherical coordinate system origin and the probe that is larger than the minimum sphere radius, *ρ*_0_, that will completely enclose the majority of the test antenna current sources [6]. This transmission formula can be inverted using the rotation group orthogonality theorem and then solved for the probe-corrected AUT transmit coefficients using the Kerns transmission formula [12]. Thus, the electric and/or magnetic fields transmitted by the test antenna in this region of space, which is also assumed to be source-free, linear, isotropic, and homogeneous, can be expressed as the linear superposition, i.e., the complex weighted sum, of spherical waves through the spherical wave summation. And, as with any form of modal expansion that is predicated on Maxwell’s equations, which, in turn, require the modal expansion region of space to be source-free, linear, isotropic, and homogeneous, there are no additional sensitivities here. Thus, so long as the normal precautions of absorber placement used in near-field measurements are followed, the method can be applied. Spherical mode coefficients (SMC) are complex numbers that are functions of frequency—the polarization index is *s*, the polar index is *n*, and the azimuthal index is *m*, such that 0 ≤ *n* ≤ ∞ and −*n* ≤ *m* ≤ *n*—which do not vary with any of the spherical scanning coordinates. The summation can, in practice, not be infinite, and instead, the maximum value for the polar summation is generally truncated to a finite positive integer number, which is large enough to enable the field to be represented precisely and accurately. Thus, it is assumed that the antenna under test (AUT) is enclosed within a sphere of radius ρ_0_ such that spherical waves of integer order *N* > *k*_0_ρ_0_, which, when taken together with an additional positive integer safety factor that depends upon the desired accuracy, represent the most complicated field structure that can be generated by the given radiator. Thus, once obtained, this finite, large set of complex coefficients can be used to accurately and very efficiently represent the radiative properties of the AUT. This makes any post-processing that acts rigorously and directly upon this set a very attractive proposition. This is precisely the approach taken by the present work. However, it is not the intent of this paper to revalidate the mode-filtering technique, as there is already a huge body of literature devoted to that cause, including many of the references included within this text and especially [6]. Instead, this paper concentrates on developing an alternative way of laying out the requisite computation that is more general than that which has come before, and it is shown to reduce to an exact numerical equivalent in the absence of evanescent fields.

The structure of the remainder of this paper is as follows. Section 2 presents a summary of the computation required by the translation of the spherical wave formula, before Section 3 presents a verification of the translation through numerical simulation. Section 4 presents an overview of the simulated experimental setup before Section 5 presents the results of the new frequency-domain spherical mode-filtering reflection suppression technique. This paper finishes with Section 6, which presents a discussion of the primary results, and provides a summary of the conclusions and the planned future work.

## 2. Translation of Spherical Waves

Let us consider two conventional right-handed coordinate systems. We may assume that these are initially coincident and synonymous. We then assume that the first, unprimed system is fixed in space with the primed system being displaced by a distance *A* in the direction specified by the conventional right-handed polar spherical angles $\theta_{0}$, $\varphi_{0}$. It can then be shown that the translation of spherical waves can be expressed, when using the shorthand formulation of [13], as a linear operation [14] so that the spherical wave function ${\vec{F}}_{smn}^{\left(c\right)}\left(r,\theta ,\varphi \right)$ can be expressed as a combination of spherical waves defined in the primed system ${\vec{F}}_{\sigma \mu \nu }^{\left(c\right)}\left(r^{\prime },\theta^{\prime },\varphi^{\prime }\right)$:

(1) $${\vec{F}}_{smn}^{\left(c=3\right)}\left(r,\theta ,\varphi \right)=\sum_{\sigma =1}^{2}\sum_{\nu =1}^{\infty }\sum_{\mu =-\nu }^{\nu }C_{\sigma \mu \nu }^{smn(c=1)}\left(\theta_{0},\varphi_{0},kA\right){\vec{F}}_{\sigma \mu \nu }^{\left(c=3\right)}\left(r^{\prime },\theta^{\prime },\varphi^{\prime }\right)$$

Here, ${\vec{F}}_{\sigma \mu \nu }^{\left(c\right)}\left(r^{\prime },\theta^{\prime },\varphi^{\prime }\right)$ are a set of spherical vector wave functions inside the primed coordinate system, with $\left(c\right)$ indicating the spherical radial function assuming the usual 1, 2, 3, and 4 numbering system introduced in [15]. Thus, we use *c* = 3 for the spherical vector wave functions before ${\vec{F}}_{\sigma \mu \nu }^{\left(c\right)}\left(r^{\prime },\theta^{\prime },\varphi^{\prime }\right)$ and then after ${\vec{F}}_{smn}^{\left(3\right)}\left(r,\theta ,\varphi \right)$ in the unprimed coordinate system once the translation operation has been applied, matching the outward traveling wave assumption from [16]. This is a very general statement, as it can be shown that this is equally valid for inward traveling waves for which (*c*) = 4. In this case, we use (*c*) = 1, i.e., Bessel functions of the first kind within the calculation of $C_{\sigma \mu \nu }^{smn(c=1)}\left(\theta_{0},\varphi_{0},kA\right)$, so that this translation can be applied to incoming *or* outgoing waves.

**Figure 1:**
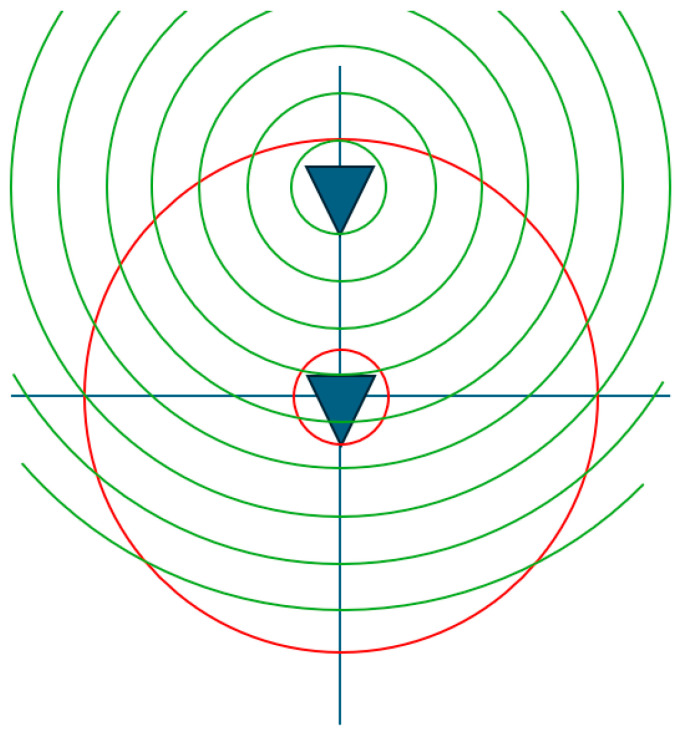
Translation of origin of the antenna as is used within this paper, where the blue triangle denotes the antenna, the red circle denotes the antenna’s minimum radial extent before and after the translation, and the green circles the phase fronts after the translation.

**Figure 2:**
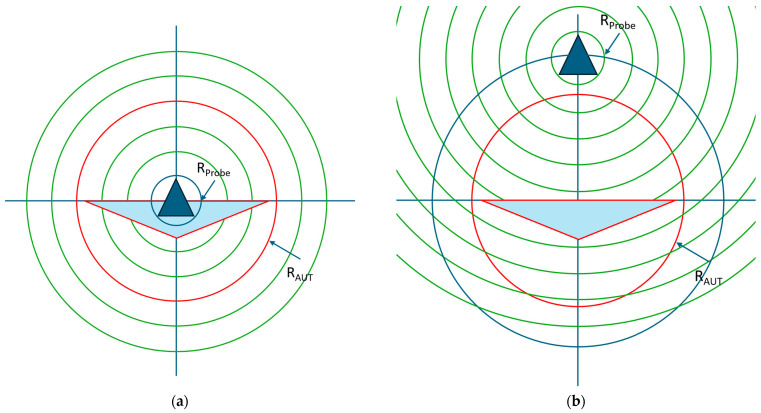
Translation of origin as used in spherical near-field antenna measurements: (**a**) untranslated case where probe and AUT coincide and (**b**) translated case where the probe is translated outside the AUT minimum radial extent. Here the blue triangle represents the probe, and the larger light blue triangle represents the AUT. Here, red and blue circles denote the minimum radial extent of the AUT and probe, respectively, and the green circles denote the wave fronts due to the probe.

It is noted here that the above translation expressed by Equation (1) is a generalization of the far-field differential phase change operation described above, which is fundamentally a *different* operation from the translation required for spherical near-field measurements, where both far-field phase *and* amplitude are affected [6]. While it is common to see in the open literature, e.g., [13,16], both translations being differentiated by explicit limitations in the form $r^{\prime }>\left|A\right|$ or $r^{\prime }<\left|A\right|$, in Appendix A, for the present work, it will be shown that there are no such limitations here except possibly those imposed by the particular application. This means that Equation (1) is valid for large magnitudes of *A* so long as it is understood that the minimum radial extent of the translated antenna also increases with *r* + *A*, and thus, the translation matrix needs to be extended accordingly to ensure sufficiently higher-order modes are available to properly represent the antenna. Similarly, the limitation of $r^{\prime }<\left|A\right|$ stems from the requirement to properly decouple the translated modes of a probe from those of the modes of the antenna under test (AUT) in a spherical near-field (SNF) measurement configuration. To better understand the difference between these cases, Figure 1 shows the translation presented in this paper where only the corresponding far-field phase distribution of the antenna pattern is affected, which is congruent to a multiplication with $e^{jk\hat{r}}$ of the far-field pattern. The minimum radial extent of the antenna increases together with its offset, as indicated by the red circles. It is noted here that the translation displaces the origin of the mode spectrum; this means that to move the AUT forward in the *z*-direction, one actually has to shift the origin of the spherical wave functions in the negative *z*-direction. Conversely, in Figure 2, the translation is *outside* the minimum radial extent of the antenna, which affects both the corresponding far-field amplitude and phase distributions. Here, Figure 2 is divided to illustrate both the untranslated and translated cases, showing its application for the special case of spherical near-field antenna measurements, where the AUT *and* the AUT radial extent remain located at the origin of the measurement coordinate system, and the probe is translated outside of the AUT radial extent. The corresponding far-field amplitude distribution of the translated antenna with increasing distance gradually narrows down until, in the limit, it becomes a single angular point on the pattern. Thus, in the limit, this corresponds to a plane wave coupling between the two spheres, thereby transitioning from a near-field to a direct far-field measurement, meaning that at certain distances the probe angular pattern essentially becomes negligible, and the compensation procedure essentially reduces to just a polarization correction.

The translation coefficients are defined as [14] and can further be expressed as

(2) $$C_{\sigma \mu \nu }^{sn(c)}\left(\theta_{0},\varphi_{0},kA\right)=\alpha \left[\delta_{s\sigma }A_{\mu \nu }^{mn}\left(\theta_{0},\varphi_{0},kA\right)+\delta_{3-s,\sigma }B_{\mu \nu }^{mn}\left(\theta_{0},\varphi_{0},kA\right)\right]$$

where α is a normalization correction factor, which is dependent upon the chosen normalization of the spherical wave expansion (SWE), and δ is the Dirac delta function [6]. Several conversions between different normalizations are reported within the open literature, with a summary being presented within (A1) of [15]. For the present work, however, the translation coefficients $A_{\sigma \mu \nu }^{smn}\left(kA\right)$ and $B_{\sigma \mu \nu }^{smn}\left(kA\right)$ are based on the spherical wave functions described by Edmonds in A1-11 of [15], while the SWE utilized to attain the spherical mode coefficients employs the normalization proposed in [13,16]. Thus, the requisite correction factor for the normalization employed here can be expressed as

(3) $$\alpha =\sqrt{\frac{\nu \left(\nu +1\right)}{n\left(n+1\right)}}.$$

with

(4) $$A_{\mu \nu }^{mn}\left(\theta_{0},\varphi_{0},kA\right)=\sum_{p=\left|n-\nu \right|}^{n+\nu }i^{-p}\left[n\left(n+1\right)+\nu \left(\nu +1\right)-p\left(p+1\right)\right]a\left(m,n,-\mu ,\nu ,p\right)z_{p}^{\left(c\right)}\left(kA\right)P_{p}^{m-\mu }\left(\cos\theta_{0}\right)e^{i(m-\mu )\varphi_{0}}$$

and

(5) $$B_{\mu \nu }^{mn}\left(\theta_{0},\varphi_{0},kA\right)=\sum_{p=\left|n-\nu \right|}^{n+\nu }i^{-p}\left[n\left(n+1\right)+\nu \left(\nu +1\right)-p\left(p+1\right)\right]\;b\left(m,n,-\mu ,\nu ,p-1\right)z_{p}^{\left(c\right)}\left(kA\right)P_{p}^{m-\mu }\left(\cos\theta_{0}\right)e^{i(m-\mu )\varphi_{0}}$$

for which

(6) $$\begin{array}{c} b(m,n,-\mu ,\nu ,p-1)\\ \;\;\;\;\;\;\;=\frac{2p+1}{2p-1}[\left(\nu -\mu \right)\left(\nu +\mu +1\right)a\left(m,n,-\mu -1,\nu ,p-1\right)\\ \;\;\;\;\;\;\;-\left(p-m+\mu \right)\left(p-m+\mu +1\right)a\left(m,n,-\mu +1,\nu ,p-1\right)+2\mu \left(p-m+\mu \right)a(m,n,-\mu ,\nu ,p-1)] \end{array}$$

Here, a(m,n,−μ,ν,p)’s are the linearization coefficients, which can be expressed in terms of Wigner 3-j symbols [15], and $z_{p}^{\left(c\right)}$ is the spherical radial function of order *p* and type (c). Furthermore, $P_{p}^{m-\mu }\left(\cos\theta_{0}\right)$ denotes the associated Legendre function of degree *p* and order (m−μ). However, given the number of factorials required, these turn out to be enormously computationally expensive to calculate directly. Fortunately, an alternative strategy may be harnessed involving the computation of these coefficients by means of efficient, stable recurrence relations [14]. Recurrence relations greatly reduce the computational complexity of calculating the coefficients, with this becoming increasingly important as the number of coefficients increases. This is especially crucial as the highest-order coefficient depends upon the frequency, minimum sphere radius, and the size of the translation. Once the scalar coefficients have been obtained, these can then be used to derive the vector addition coefficients. When taking this approach, the addition coefficients can be written as follows:

(7) $$\begin{array}{c} A_{\mu \nu }^{mn}\left(\theta_{0},\varphi_{0},kA\right)=\beta_{\mu \nu }^{mn}\left(\theta_{0},\varphi_{0},kA\right)+kA\sin\theta_{0}\frac{e^{-i\varphi_{0}}}{2\left(\nu +1\right)}\sqrt{\frac{\left(\nu -\mu +2\right)\left(\nu -\mu +1\right)}{\left(2\nu +1\right)\left(2\nu +3\right)}}\beta_{\mu -1,\nu +1}^{mn}\left(\theta_{0},\varphi_{0},kA\right)\\ \;\;\;\;\;\;\;-kA\sin\theta_{0}\frac{e^{-i\varphi_{0}}}{2\nu }\sqrt{\frac{\left(\nu +\mu -1\right)\left(\nu +\mu \right)}{\left(2\nu -1\right)\left(2\nu +1\right)}}\beta_{\mu -1,\nu -1}^{mn}\left(\theta_{0},\varphi_{0},kA\right)\\ \;\;\;\;\;\;\;-kA\sin\theta_{0}\frac{e^{i\varphi_{0}}}{2\left(\nu +1\right)}\sqrt{\frac{\left(\nu +\mu +2\right)\left(\nu +\mu +1\right)}{\left(2\nu +1\right)\left(2\nu +3\right)}}\beta_{\mu +1,\nu +1}^{mn}\left(\theta_{0},\varphi_{0},kA\right)\\ \;\;\;\;\;\;\;+kA\sin\theta_{0}\frac{e^{i\varphi_{0}}}{2\nu }\sqrt{\frac{\left(\nu -\mu \right)\left(\nu -\mu -1\right)}{\left(2\nu -1\right)\left(2\nu +1\right)}}\beta_{\mu +1,\nu -1}^{mn}\left(\theta_{0},\varphi_{0},kA\right)\\ \;\;\;\;\;\;\;+kA\sin\theta_{0}\frac{1}{\nu +1}\sqrt{\frac{\left(\nu +\mu +1\right)\left(\nu -\mu +1\right)}{\left(2\nu +1\right)\left(2\nu +3\right)}}\beta_{\mu ,\nu +1}^{mn}\left(\theta_{0},\varphi_{0},kA\right)\\ \;\;\;\;\;\;\;+kA\sin\theta_{0}\frac{1}{\nu }\sqrt{\frac{\left(\nu +\mu \right)\left(\nu -\mu \right)}{\left(2\nu -1\right)\left(2\nu +1\right)}}\beta_{\mu ,\nu -1}^{mn}\left(\theta_{0},\varphi_{0},kA\right) \end{array}$$

and

(8) $$\begin{array}{c} B_{\mu \nu }^{mn}\left(\theta_{0},\varphi_{0},kA\right)=kA\cos\theta_{0}\frac{i\mu }{\nu \left(\nu +1\right)}\beta_{\mu \nu }^{mn}\left(\theta_{0},\varphi_{0},kA\right)\\ \;\;\;\;\;\;\;+\frac{ikA\sin\theta_{0}}{2\nu \left(\nu +1\right)}[\sqrt{\left(\nu -\mu \right)\left(\nu +\mu +1\right)}e^{i\varphi_{0}}\beta_{\mu +1,\nu }^{mn}\left(\theta_{0},\varphi_{0},kA\right)\\ \;\;\;\;\;\;\;+\sqrt{\left(\nu +\mu \right)\left(\nu -\mu +1\right)}e^{-i\varphi_{0}}\beta_{\mu -1,\nu }^{mn}\left(\theta_{0},\varphi_{0},kA\right)] \end{array}$$

Here, Equations (7) and (8) refer to the calculation of the scalar translation matrix, which is in the form of $\beta_{\mu \nu }^{mn}\left(\theta_{0},\varphi_{0},kA\right)$, for which recurrence relations also exist and are developed in [17]. Here, the starting values for *n* = 0, *m* = 0, i.e., $\beta_{\nu \mu }^{00}\left(\theta_{0},\varphi_{0},kA\right)$ can be easily derived from the scalar wave equation using

(9) $$\beta_{\nu \mu }^{00}\left(\theta_{0},\varphi_{0},kA\right)={\left(-1\right)}^{\mu +\nu }\sqrt{4\pi }Y_{\nu ,-\mu }\left(\theta_{0},\varphi_{0}\right)z_{n}^{\left(c\right)}\left(kr\right)$$

Here, $z_{n}^{\left(c\right)}$ is the spherical radial function of order *n* and type (c).

(10) $$Y_{n,m}\left(\theta_{0},\varphi_{0}\right)={\left(-1\right)}^{m}{\left[\frac{\left(n-m\right)!2n+1}{\left(n+m\right)!4\pi }\right]}^{\frac{1}{2}}P_{n}^{\left|m\right|}\left(\cos\theta_{0}\right)e^{im\varphi_{0}}$$

where ${\bar{P}}_{n}^{\left|m\right|}\left(\cos\theta \right)$ is the associated Legendre function. This calculation also makes use of the standard relation $Y_{n,-m}\left(\theta_{0},\varphi_{0}\right)=\;{\left(-1\right)}^{m}Y_{n,m}^{*}\left(\theta_{0},\varphi_{0}\right)$ [17]. Then, $\beta_{\mu \nu }^{nn}$ may be expanded using the relation

(11) $$b_{nn}^{+}\beta_{\nu \mu }^{n+1,n+1}=b_{\nu -1,\mu -1}^{+}\beta_{\nu -1,\mu -1}^{nn}+b_{\nu +1,\mu -1}^{-}\beta_{\nu +1,\mu -1}^{nn}$$

where

(12) $$b_{nm}^{+}={\left[\frac{\left(n-m\right)\left(n-m-1\right)}{\left(2n+1\right)\left(2n-1\right)}\right]}^{\frac{1}{2}}$$

and

(13) $$b_{nm}^{-}={\left[\frac{\left(n+m+2\right)\left(n+m+1\right)}{\left(2n+1\right)\left(2n+3\right)}\right]}^{\frac{1}{2}}$$

The recurrence relation for $\beta_{\mu \nu }^{n+1,m}$ is derived from the $\beta_{\mu \nu }^{nn}$ values, where the starting points are *m* = *n* so that the requisite recurrence relations are

(14) $$a_{nm}^{+}\beta_{\nu \mu }^{n+1,m}=-a_{nm}^{-}\beta_{\nu \mu }^{n-1,m}+a_{\nu -1,\mu }^{+}\beta_{\nu -1,\mu }^{nm}+a_{\nu +1,\mu }^{-}\beta_{\nu +1,\mu }^{nm}$$

where

(15) $$a_{nm}^{+}={\left[\frac{\left(n+m+1\right)\left(n-m+1\right)}{\left(2n+1\right)\left(2n+3\right)}\right]}^{\frac{1}{2}}$$

and

(16) $$a_{nm}^{-}={\left[\frac{\left(n+m\right)\left(n-m\right)}{\left(2n+1\right)\left(2n-1\right)}\right]}^{\frac{1}{2}}$$

Lastly, $\beta_{\mu \nu }^{n,-m}$ is derived from $\beta_{\mu \nu }^{n,-m}$ using

(17) $$\beta_{\nu \mu }^{n,-m}={\left(-1\right)}^{\mu +m}\beta_{\nu ,-\mu }^{nm}$$

Thus, using Equation (1), a translation can be applied in *any* direction and in either sense, where the displacement is defined as $\underline{r}=\theta_{0}\underline{\hat{\theta }}+\varphi_{0}\underline{\hat{\varphi }}+kA\underline{\hat{r}}$, and where the magnitude of the displacement |*r*| can be larger *or* smaller than the minimum sphere radius.

The memory consumption of the final translation matrix depends on the number of input and output modes considered. If the AUT at the input is displaced, the occupied MRE is expected to be larger than the AUT itself, which, after translation back to the origin, is reduced. The size of the translation matrix can be written as follows, assuming for each point P a double-precision complex number:

(18) $$P=J_{in}\cdot J_{out}=2N_{in}\left(N_{in}+2\right)\cdot 2N_{out}\left(N_{out}+2\right)$$

with

(19) $$N=\frac{2\pi a}{\lambda },$$

where a is the MRE, and λ is the wavelength.

For the pre-calculation of $\beta_{\mu ,\nu }^{mn}$, however, a matrix of the following size is required as well, which is about half the size of the translation matrix:

(20) $$P=\left(\left(N_{in}+N_{out}\right)\left(N_{in}+N_{out}+2\right)+1\right)\cdot \left(N_{out}\left(N_{out}+2\right)+1\right)$$

In principle, there is no practical numerical limitation to the maximum number of *N*-modes that can be used with the recursive formulations to determine the translation matrix. However, the direct calculation of the Legendre polynomials currently utilized has a higher-order limit of approximately *N* = 85, although when required, this can be easily extended using the Symbolic Math Toolbox or by adopting alternative recursion formulae that are available in the open literature [15].

## 3. Verification of the Translation of Origin Formula

**Figure 3:**
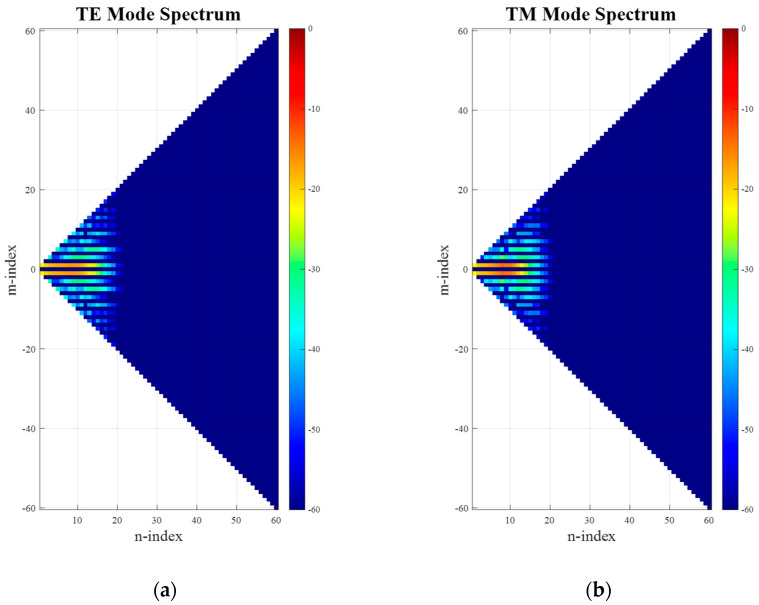
Mode spectra of simulated SGH: (**a**) TE modes; (**b**) TM modes.

In order to verify the translation algorithm, a standard gain horn (SGH) pattern has been simulated using its amplitude and phase distributions sampled across a small surface distributed on the *xy*-plane at *z* = 0, from which, using the Kirchhoff–Huygens formula [6], the full far-fields may be determined. In so doing, it is understood that the resulting far-field pattern origin is not coincident and synonymous with the antenna pattern phase center. This is not a restriction for the purpose of the following verification. The far electric fields can then be expanded onto a set of spherical modes to yield the spherical mode spectrum shown in Figure 3. Here, it can be clearly seen that the minimum radial extent is limited such that the lowest *circa* 25 modes contain the majority of the power radiated by the antenna and should be sufficient to describe the antenna far-field pattern, as in this case of *n_max_* = *k*_0_*a* + 10 = 25, where 10 is a typical empirical factor that is usually added for problems of this electrical size and which depends upon the desired accuracy, cf. [6,16], especially [18]. Next, we apply a translation to the AUT in the z-axis using the conventional approach of adjusting the far-field phase function. Thus, as a second step, on the far-field pattern, a phase-shift is applied, which is a function of the polar pattern angle θ, the free-space propagation constant *k*_0,_ and the displacement distance *Offset*. This phase shift represents the physical movement of the antenna in the *z*-direction away from its phase center and measurement origin. This can therefore be expressed as

(21) $$w_{\mathrm{offset}}\left(\theta ,\varphi ,\chi \right)=w_{0}\left(\theta ,\varphi ,\chi \right)e^{ik\cos\left(\theta \right)\mathrm{Offset}}.$$

where $w_{0}$ is the far-field measurement of the SGH when at the origin, and $w_{\mathrm{offset}}$ is the representation of the measurement with the offset applied.

**Figure 4:**
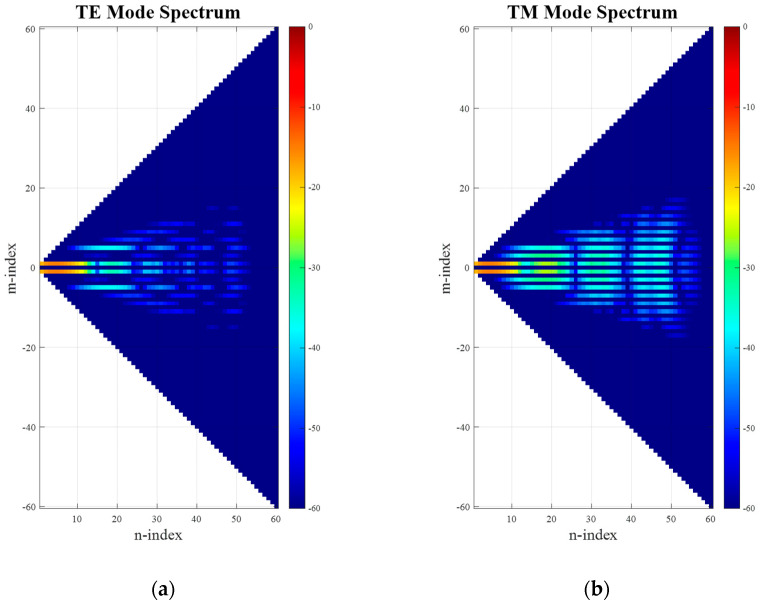
Mode spectra of simulated SGH with added offset: (**a**) TE modes; (**b**) TM modes.

After applying an SWE to the far-field pattern of the translated antenna (including the phase shift), the resulting spherical mode distribution can be seen in Figure 4. While comparing Figure 3 and Figure 4, it is clear that the occupied mode space in *N* can be seen to have significantly increased, extending up to the full calculated space of 60 modes. This is expected, as now *n_max_* = *k*_0_(*a* + *Offset*) + 10 = 60. However, due to the purely *z*-directed translation direction, the pattern symmetry is not broken, and thus the distribution width in *M*-direction has been preserved.

**Figure 5:**
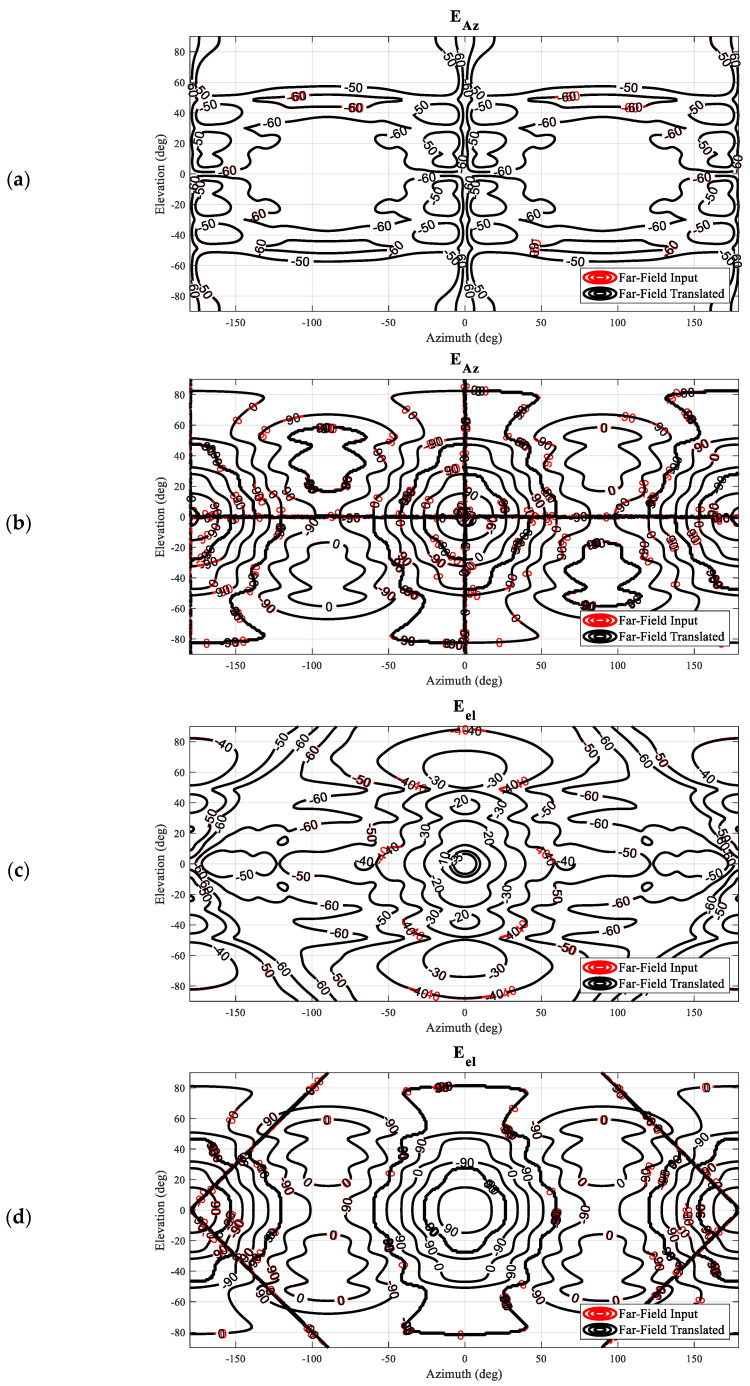
Far-field pattern comparison between simulated SGH and back translation from the offset: (**a**) Amplitude of the azimuth component. (**b**) Phase of the azimuth component. (**c**) Amplitude of the elevation component. (**d**) Phase of the elevation component.

Next, we may translate the antenna back to its original position by utilizing the vector addition theorem presented above to implement an equivalent translation directly upon the SMCs. In the case that the mode translation operation works correctly for this case, the resulting far-field amplitude and phase patterns should agree exactly with the initial starting data. In Figure 5, comparison contour plots of the simulated antenna (input) and the back-translated patterns are shown tabulated on a regular azimuth-over-elevation grid, denoted by red and black contours, respectively. Here, it can be seen that for both field components, the amplitude patterns (a) and (c) are nearly indistinguishable from one another. The phase distribution of the elevation field component, which represents the co-polar pattern, is found to have an excellent match as well, while the cross-polar azimuth field component shows a little larger difference between the input and the translated data. One has, however, to keep in mind that the respective amplitudes are more than 50 dB below the pattern peak, and most likely the differences are caused by the higher-order mode cut-off of the mode spectrum of the shifted pattern, as well as numerical truncation and rounding, rather than due to the translation operation itself. That being said, the co-polar and cross-polar amplitude and phase patterns are all in such good agreement that each of the contours overlays precisely, with even the positions of the contour labels coinciding, which is a very encouraging result indeed.

Based on the performed comparisons shown in Figure 5, it can be concluded that the translation of origin described can be used as an alternative to the far-field pattern-based displacement operation, which multiplies the far-field antenna pattern function with a complex exponential, $e^{jk\hat{r}}$, and filters out any evanescent fields. And, as has been verified previously, cf. [19], the mode translation can be applied in any direction and not merely along the *z*-axis. Appendix B below contains a further example of the verification of a general translation that was specifically *not* aligned with the *z*-axis.

## 4. Experimental Setup of the Mode Filtering

**Figure 6:**
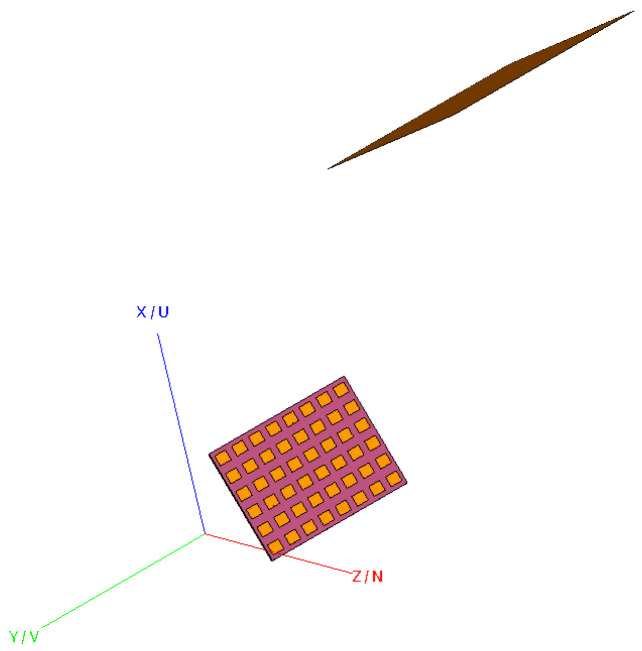
The “measurement” setup. The AUT is on the left, and the scatterer is on the top of the figure.

To further validate the application of the generalized vector addition theorem to the mode-filtering-based reflection suppression, a numerical model has been created using the proprietary full-wave three-dimensional computational electromagnetic solver Altair Feko [20]. To make the simulation sufficiently realistic, a model of a 48-element planar array antenna was simulated and used as an AUT [21]. The spherical near-field measured pattern of the AUT was then perturbed with the inclusion of a perfect electric conducting (PEC) square plate, which was included to create the requisite complex environment and provide the parasitically coupled scatterer. Figure 6 shows the experimental configuration in the modeling software. By stepping the orientations of the model of the AUT through a sequence of rotations, a measurement using a traditional “roll-over-azimuth” positioning system [6] was simulated. Figure 6 shows a randomly chosen example where the AUT has been rotated to the virtual measurement angle of θ=30°. In this particular simulation, the AUT was located displaced by 360 mm from the origin of the simulated spherical “measurement” setup, in *z*-direction of the AUT coordinate system. On the top-right-hand side, a large PEC scatterer can be seen. Here, the center of the reflecting plate is positioned 1000 mm above and right from the global coordinate system origin and oriented at approximately 25° to the *yz*-plane, which results in a maximum effect in disturbing the far-field pattern of the AUT. It is further emphasized that the location and orientation of the plate remain statically located with respect to the global coordinate system, which mirrors the practical case of a scatterer that is located at a fixed position within the test chamber. The amplitude and phase distribution of the AUT was previously described in reference [21]. While the excitation is not especially important for the present work, the general complex nature of the field distribution makes it far more attractive than a simple analytical field such as that used above to confirm the validity and applicability of the translation algorithm itself.

When constructing a measurement simulation such as that needed here, one has to keep in mind that whilst the simulation software does allow the user to sample the radiated field from some given radiating structure in three-dimensional space quite easily. In the present case, where the AUT has to be rotated through a full range of $\left(\theta ,\varphi \right)$ orientations of the virtual “model-tower” spherical positioning system [6], the scatterer has to retain its position and orientation relative to the sampling near-field probing point. Thus, for each unique spherical near-field position, the model must be simulated separately, after which each time a single near- or far-field point of the model can be sampled in the range “boresight” direction at θ = 0°, φ = 0° in the global coordinate system. Thus, when using a 3° angular data point spacing in both θ and φ directions, which is required by the spherical sampling theorem for this size problem and test frequency, this leads to a total of 7320 points to be “measured”, i.e., 7320 individual, full-wave method-of-moment (MoM) simulations. Although there are a great many different computational electromagnetic (CEM) simulation methods that could have been harnessed for the purpose of constructing this simulation, MoM was chosen as it encapsulates all of the complex scattering properties of this problem, which avoids the need to mesh large regions of vacuum, which would have otherwise greatly increased the problem size and made this already large and computationally expensive simulation significantly more challenging.

## 5. Application of the Generalized Vector Addition Theorem in Mode Filtering

**Figure 7:**
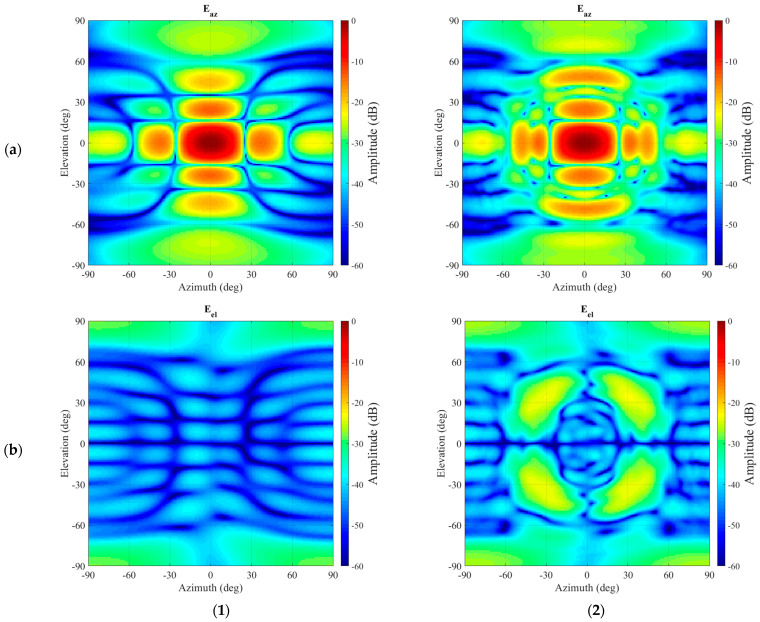
Azimuth-over-elevation projection of the far-field of the antenna without (**1**) and with (**2**) interference present: (**a**) Amplitude of the azimuth polarization component. (**b**) Amplitude of the elevation polarization component.

The full-wave CEM simulation described above was then used to provide the data needed to carefully verify the new processing algorithm. Thus, for the present case, a spherical near-field measurement was simulated with a measurement radius of 2.0 m. As a reference, a second set of simulated “measured” spherical near-field data was attained without having the scatterer present. This is a conventional simulation that can be obtained using a simple model of the test antenna in isolation. Both SNF data sets were then processed using a standard spherical near-field to far-field transformation (SNFFFT) [6]. This allows the effect that the scatter has on the far-field pattern of the particular AUT to be clearly seen. Given the large electrical size of the scatterer, the effect on the pattern is intended to be significant, and this can be clearly seen by examining the contour plots of the far electric fields, which have been tabulated on a regular azimuth-over-elevation projection and resolved into a commensurate Ludwig II azimuth over elevation polarization basis [6]. These plots can be seen in Figure 7. While the boresight peak seems largely unaffected, for azimuth and elevation angles of *circa* 30° and greater, the differences in spurious interfering side-lobes from the parasitically coupled scatterer become increasingly evident in both the principal and cross-polarised field components. Here, the azimuth field component contains the co-polarization signal, and the elevation field component contains the cross-polarization signal. While clearly the disturbance is visible in the co-polarization component, it is very pronounced in the cross-polarization.

Utilizing the perturbed dataset that was contaminated with the PEC interference, the novel spherical mode-filtering reflection suppression algorithm was applied. The revised spherical mode-filtering reflection suppression algorithm relies upon the same physical phenomena and theory as prior implementations. However, it consists of fewer, more generally applicable steps when compared with those presented in earlier publications, cf. [6], for example. The generalized revised approach can be summarized as follows:

1. The antenna under test is displaced from the origin of the measurement sphere in a known direction and distance, $\underline{r}=\theta_{0}\underline{\hat{\theta }}+\varphi_{0}\underline{\hat{\varphi }}+kA\underline{\hat{r}}$;
2. A spherical (near-field) measurement is performed with a sufficiently fine angular sampling resolution to provide the unaliased mode spectrum required to describe the offset antenna pattern function;
3. A spherical wave expansion is used to compute the spherical mode coefficient of the measurement;
4. Probe pattern compensation is applied to the computed set of spherical modes (this was not required in this case, as an ideal electric Hertzian dipole probe was used within the SNF CEM simulation);
5. The spherical mode coefficients are translated to the origin by means of the translation matrix described above;
6. The translated set of SMCs is filtered to extract the deleterious scattered modes, cf. [6];
7. Finally, a spherical wave summation is applied to attain the mode-filtered far-field pattern.

The calculation of the translation matrix used in step 5 for the given problem takes approximately 8 s, which, however, can be precalculated and reused for subsequent antenna measurements having the same offset and frequency. This can be further reduced if the highest-order N-mode in the output spectrum after translation is reduced (which is expected as the AUT moves towards the phase center). In cases of varying antenna sizes, a larger (up to higher-order N-modes) translation matrix can be precalculated, and for smaller antennas, only a subset of the matrix is utilized. Assuming a precalculated translation matrix, step 5 takes only 0.1 s versus 0.2 s for the same general operation of the original algorithm. Note, the degree of orthogonalization attained between antenna and parasitically coupled scattering modes is largely dependent upon the magnitude of the AUT offset and is largely independent of the measurement radius [5,6].

**Figure 8:**
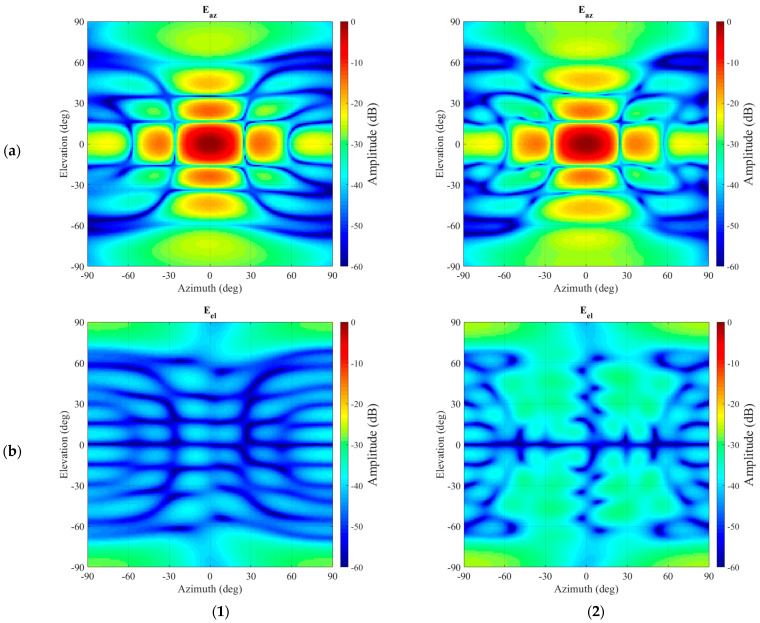
Far-field comparison between reference pattern without interference present (left hand column **1**) and the filtered pattern (right hand column **2**). Amplitude of the azimuth polarization component (**a**) and amplitude of the elevation polarization component (**b**).

Figure 8 presents a comparison between the resulting far-field pattern after the application of the mode-filtering and the unperturbed far-field pattern. It can be seen, especially while comparing Figure 7 and Figure 8, that even though some residue of the unwanted, originally very significant interference persists in the far-field pattern, the vast majority of the spurious side-lobes in the azimuth (co-) polarization have been largely attenuated with the resulting pattern being in far closer agreement with the unperturbed reference far-field antenna pattern. The improvements in the cross-polarization are more difficult to assess, considering the relatively low signal levels contained within the reference pattern. However, the large lobes that were initially evident in the intercardinal region at *circa* 30° have been very effectively suppressed.

**Figure 9:**
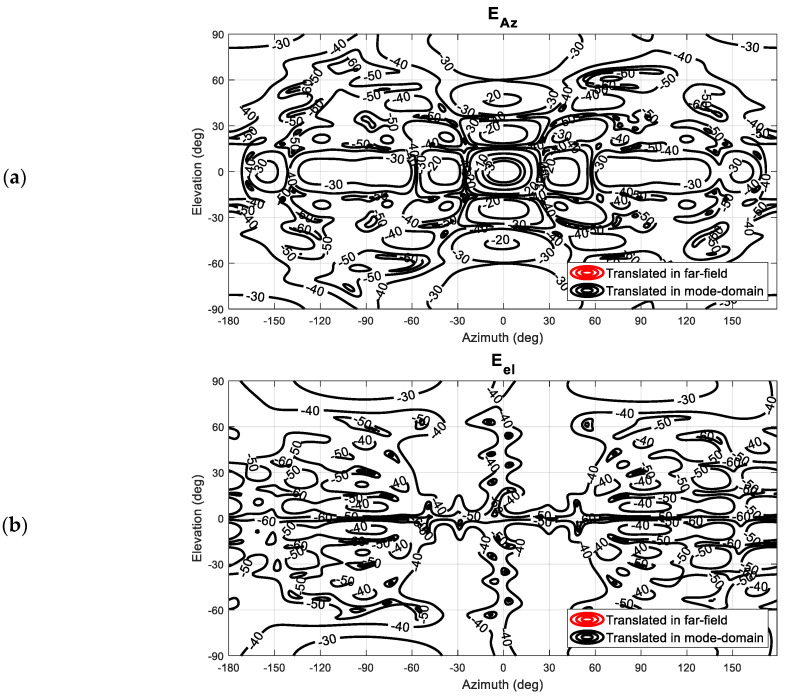
Far-field-based mode filtering compared to spherical wave translation-based mode filtering: (**a**) Amplitude of the azimuth polarization component. (**b**) Amplitude of the elevation polarization component.

**Figure 10:**
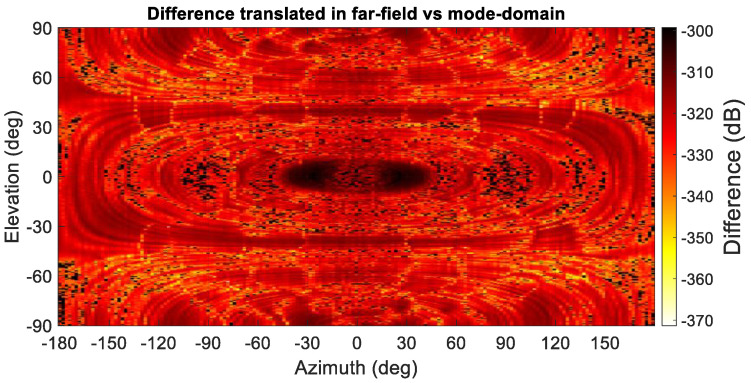
Difference between the far-field-based mode filtering and the spherical wave translation-based mode filtering.

By way of a further examination of the validity and reliability of the revised mode-filtering algorithm, a careful comparison against the traditional approach was undertaken. While the presented method in itself is expected only to attain significant benefits in those cases where the evanescent modes of the antenna in the near-field become more significant or when attempting to extract isolated single element radiating properties from array antenna measurements, which, it is important to note, was one of the primary applications and motivation of the spherical mode-filtering approach [6], it is important to verify equivalence with the existing approach within the region of validity. Thus, although no improvements when compared with the existing far-field-based method are anticipated, it is important to investigate whether equally valid results are obtained. Therefore, the perturbed dataset was also transformed into the true far-field so that the test antenna could be mathematically translated back into the origin using the standard differential phase change approach. These far-fields were then used to compute the equivalent SMC, which were then filtered using the same cut-off as was used when processing with the new translation spherical mode-filtering reflection suppression was applied. This result can be seen compared in Figure 9. Here, one can observe that both filtering methods provide nearly identical results. By way of a further comparison, the dB difference level [6] of both filtered far-field patterns is compared in Figure 10, where their degree of agreement is nearing the limit of double-precision arithmetic.

Furthermore, Figure 10 presents a false color checkerboard plot of the dB difference level between the results of the mode-filtering algorithms, indicating the extremely encouraging agreement attained. Thus, although the mode-filtering results demonstrate that not all of the parasitically coupled spurious scattered field has been extracted from the grossly contaminated simulated SNF measurement, the new algorithm is equally effective as the traditional approach, only with fewer transform operations being required and with the retention of any evanescent components.

Lastly, it is important to note that in this area of application, we are always translating AUT modes back towards the origin and *not* reducing the total number of *N*-modes. Thus, the mode-truncation effect is equal to the well-known truncation rules for spherical modes [6] when considering an AUT measured at an offset position. However, for the alternative case where one is translating away from the origin (to a larger distance), then the output *N* can increase; however, this too would be determined equivalently to the case where the AUT was measured offset at that position.

## 6. Discussion and Conclusions

This paper has introduced a new data processing algorithm that is equivalent to the traditional frequency-domain spherical mode-filtering-based scattering suppression technique that no longer requires spherical near-field data to be transformed into the asymptotic true far-field to implement the requisite translation of origins that is necessary to separate antenna modes from parasitically coupled scattering modes prior to mode filtering. This study utilized an extensive full-wave CEM simulation to provide data necessary to enable the very precise verification and validation of the new algorithm. The generalized spherical addition theorem was used to implement the translation of the test antenna in any direction and by a displacement that could be larger, smaller, or equal to the MRE, with this operation being applied to the antenna when represented as a finite set of spherical mode coefficients and being very general since it is *not* limited to purely considering the first-order azimuthal modes, but rather is equally valid for all azimuthal modes for which |*m*| ≤ *n*. The success of the revised, streamlined spherical mode-filtering-based reflection suppression algorithm was verified against a traditional far-field phase modification-based equivalent, with agreement being attained to within the limits of double-precision arithmetic. This is important since it provides valuable further verification for *both* approaches and is the first time that this sort of validation has been reported within the open literature. The planned future work is to include further verification of the translation operation in non-longitudinal directions.

## Figures and Tables

**Figure A1:**
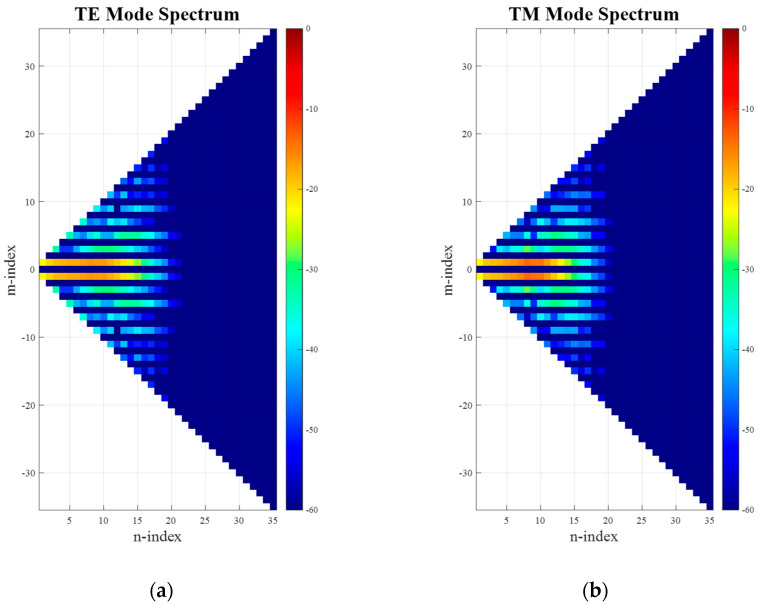
Mode spectra of simulated SGH without offset: (**a**) TE modes; (**b**) TM modes.

**Figure A2:**
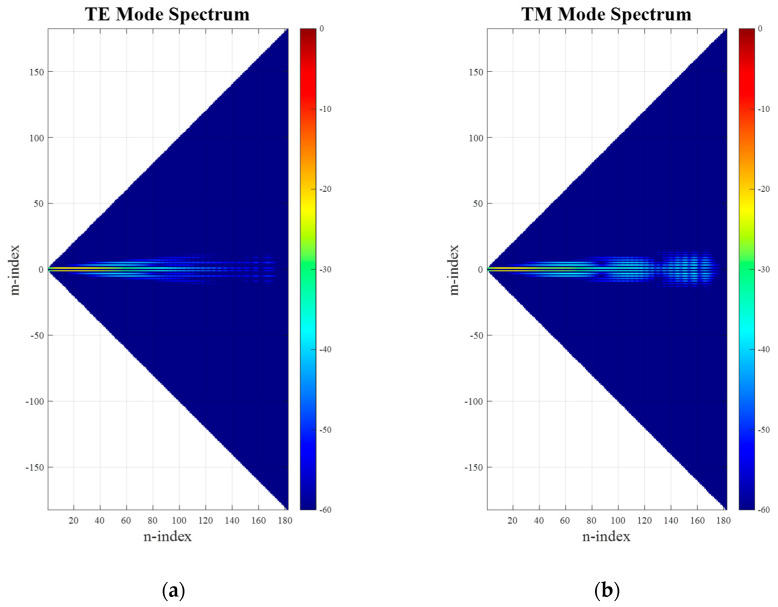
Mode spectra of simulated SGH at 1 m offset: (**a**) TE modes; (**b**) TM modes using Equation (1).

**Figure A3:**
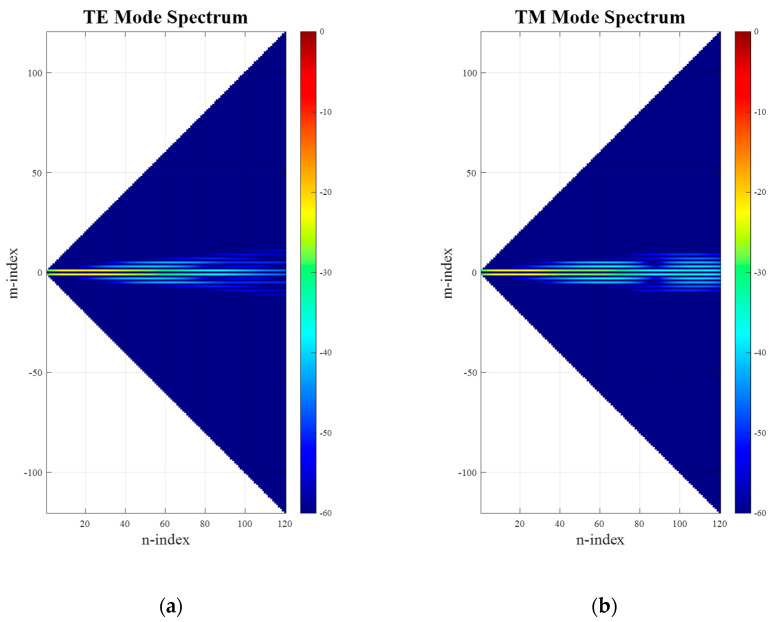
Mode spectra of simulated SGH at 1 m offset: (**a**) TE modes; (**b**) TM modes using the translation used in spherical near-field antenna measurements.

**Figure A4:**
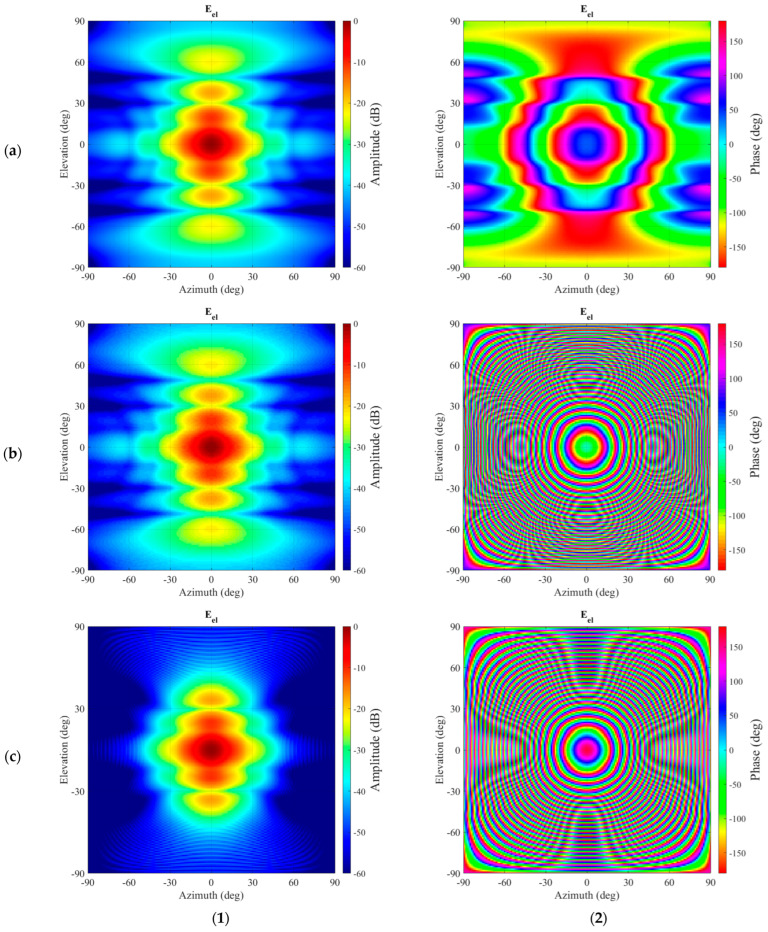
Far-field pattern amplitude (**1**) and phase (**2**) of the elevation field component comparing untranslated (**a**) with translated using Equation (1) (**b**) and translation for spherical near-field antenna measurements (**c**).

**Figure A5:**
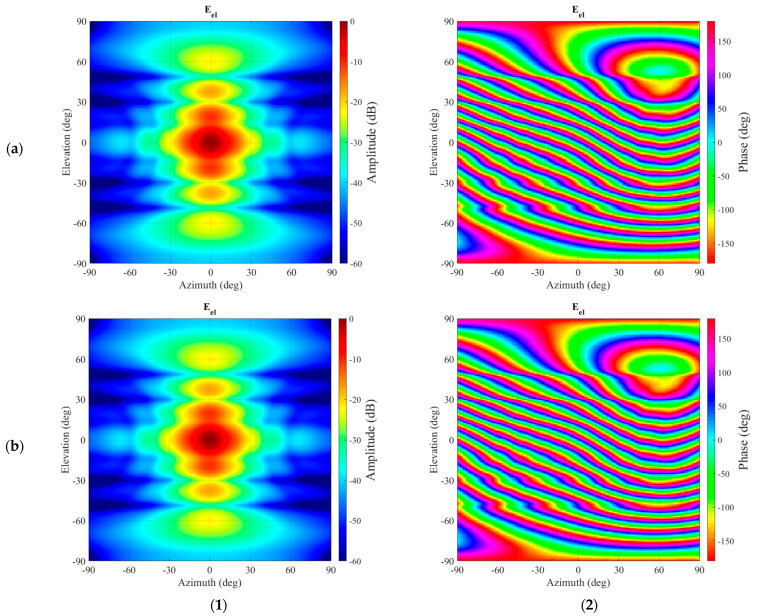
Far-field pattern amplitude (**1**) and phase (**2**) of the elevation field component comparing translation in the far-field (**a**) with the translation using Equation (1) (**b**).

## Data Availability

The original contributions presented in this study are included in the article. Further inquiries can be directed to the corresponding author.

## Abbreviations

The following abbreviations are used in this manuscript:

AUT	Antenna Under Test
CEM	Computational Electromagnetic
MoM	Method of Moments
MRE	Maximum Radial Extent
PEC	Perfect Electric Conductor
SGH	Standard Gain Horn
SMC	Spherical Mode Coefficients
SNFFFT	Spherical Near-Field to Far-Field Transform
SWE	Spherical Wave Expansion
SWS	Spherical Wave Summation

## Appendix A

As was noted above, and contrary to much of the open literature, here we have made the assertion that for the generalized antenna translation presented herein, no such limitation exists on the maximum displacement, providing the mode spectrum of the translated antenna is permitted to grow according to the increase in MRE, due to the change in the virtual distance to the origin of the spherical mode functions. To validate these assertions, a standard gain horn (SGH) pattern was simulated using its typical amplitude and phase distribution across a small surface distributed on the *xy*-plane at *z* = 0, for which, using the Kirchhoff–Huygens formula [6], the near-field has been determined at a distance of 600 mm. The MRE of the SGH was conservatively estimated as being 140 mm. Based on the well-known estimation of the required modal bandwidth N=kr+10 [6], it can be estimated that N=35 should be a sufficiently large maximum to describe the radiation pattern of this SGH using a finite set of spherical mode coefficients. Based on this, a simulated SNF measurement having a 3° angular resolution is sufficient for the near-field to far-field transformation to provide reliable far-fields. The resulting spherical mode spectrum is shown in Figure A1. Here, it can be seen that a maximum of 35 polar modes is more than enough to describe the SGH pattern.

Next, the antenna was mathematically translated in the positive *z*-direction by a distance of 1000 mm. The resulting minimum radial extent increases with the displacement of the antenna by approximately 1000 mm, and as a result, the required model bandwidth increases to approximately N=182. Thus, when the translation matrix is computed, it is configured to have only N=35 modes on the right-hand side, while containing N=182 on the left-hand side of Equation (1). The resulting spherical mode spectrum is shown in Figure A2. Here, and as expected from the *z*-directed translation, the rotational symmetry of the antenna pattern has been retained under this isometric translation, and thus the spherical mode spectrum is only increased towards higher-order modes in the polar *N* axis. For completeness, Figure A3 contains equivalent mode spectra for thew case where the radiator has been displaced using the translation that is utilized within standard spherical near-field to far-field transform. By comparing Figure A2 and Figure A3 clear differences in the respective mode spectra are clear.

After evaluating the spherical wave summation, in the far-field, the amplitude patterns of the simulated SGH were compared to those of the translated SGH, which are shown in Figure A4(a1,b1). Here, it can be seen that the differences between the patterns are negligible. While the far-field amplitude pattern has remained constant, the expected addition of the phase taper to the far-field pattern is evaluated as well and shown in Figure A4(a2,b2) for the elevation field component, which entails the co-polarization of the pattern. The phase patterns, however, show, as expected, a strong phase taper for the translated antenna.

Next, the same SGH was translated using the usual translation functions applied in spherical near-field antenna measurements, where it is expected that the probe pattern narrows down to a plane wave with increasing distance. Here, both amplitude and phase patterns are affected by the translation, which is shown in Figure A4(c1,c2). Here, we have illustrated important features of these distinct operations that are not widely recognized and have not previously been highlighted within the open literature.

## Appendix B

While thus far, only z-directed translations have been shown, an important aspect of the translation matrix approach is the capability of translation in an arbitrary direction. In the following experiment, a translation offset of $\left[x,y,z\right]=[0.1,\;0.2,\;0.1]$ is used to translate the same AUT as used within the previous appendix away from its measured origin. The translation is then compared to the equivalent translation applied directly to the far-field pattern. Figure A5a shows the elevation component in amplitude (1) and phase (2) of the direct far-field translation and is to be compared with the matrix translation shown in Figure A5b. From inspection, we see that the agreement is extremely encouraging, with an RMS dB difference level of −191 dB below the amplitude patterns, which far exceeds the accuracy required by any practical measurement. Finally, it is worth noting that although in principle it is possible to use a pure *z*-translation in combination with a pre-rotation and a post-rotation to achieve the same generalized translation operation, that too must be performed using the same z-translation operation developed within this paper, and not in the form of a translation ordinarily utilized in probe-compensated spherical near-field to far-field transforms (cf. Appendix A above for a detailed treatment of this difference).
